# Zoom behavior during visual search modulates pupil diameter and reflects adaptive control states

**DOI:** 10.1371/journal.pone.0282616

**Published:** 2023-03-09

**Authors:** Tad T. Brunyé, Trafton Drew, Kathleen F. Kerr, Hannah Shucard, Kate Powell, Donald L. Weaver, Joann G. Elmore

**Affiliations:** 1 Center for Applied Brain and Cognitive Sciences, Tufts University, Medford, MA, United States of America; 2 Department of Psychology, University of Utah, Salt Lake City, UT, United States of America; 3 Department of Biostatistics, University of Washington, Seattle, WA, United States of America; 4 Department of Pathology, University of Vermont and Vermont Cancer Center, Burlington, VT, United States of America; 5 David Geffen School of Medicine, Department of Medicine, University of California, Los Angeles, CA, United States of America; Albanian University, ALBANIA

## Abstract

Adaptive gain theory proposes that the dynamic shifts between exploration and exploitation control states are modulated by the locus coeruleus-norepinephrine system and reflected in tonic and phasic pupil diameter. This study tested predictions of this theory in the context of a societally important visual search task: the review and interpretation of digital whole slide images of breast biopsies by physicians (pathologists). As these medical images are searched, pathologists encounter difficult visual features and intermittently zoom in to examine features of interest. We propose that tonic and phasic pupil diameter changes during image review may correspond to perceived difficulty and dynamic shifts between exploration and exploitation control states. To examine this possibility, we monitored visual search behavior and tonic and phasic pupil diameter while pathologists (N = 89) interpreted 14 digital images of breast biopsy tissue (1,246 total images reviewed). After viewing the images, pathologists provided a diagnosis and rated the level of difficulty of the image. Analyses of tonic pupil diameter examined whether pupil dilation was associated with pathologists’ difficulty ratings, diagnostic accuracy, and experience level. To examine phasic pupil diameter, we parsed continuous visual search data into discrete zoom-in and zoom-out events, including shifts from low to high magnification (e.g., 1× to 10×) and the reverse. Analyses examined whether zoom-in and zoom-out events were associated with phasic pupil diameter change. Results demonstrated that tonic pupil diameter was associated with image difficulty ratings and zoom level, and phasic pupil diameter showed constriction upon zoom-in events, and dilation immediately preceding a zoom-out event. Results are interpreted in the context of adaptive gain theory, information gain theory, and the monitoring and assessment of physicians’ diagnostic interpretive processes.

## Introduction

Pupil diameter is influenced by both external and internal states, constantly adapting to environmental lighting conditions, focal distance, and arousal and mental workload [1]. In relatively low-light, narrow depth of field, and higher arousal or mental effort conditions, pupil diameter tends to increase [1, 2]. Many pupil responses are reflexive, controlled by subcortical mechanisms that quickly adapt pupil diameters as a function of contextual factors; other pupil responses, however, are controlled by cortical mechanisms that influence pupil diameter in a top-down manner. For example, early research demonstrated increases in pupil diameter (dilation) in response to difficult mental arithmetic [3] and increasing working memory load [4], leading scientists to propose that task-evoked pupil responses are a reliable physiological measure of mental processing load [5] or load on attentional capacity [6].

While pupil diameter is now generally accepted as a measure of load on attention and memory in controlled laboratory settings, its physiological basis remains somewhat elusive [7]. Pupil diameter is controlled by a parasympathetic constriction pathway connecting the retina to the iris sphincter muscle, and a sympathetic dilation pathway connecting the hypothalamus, locus coeruleus (LC), and the iris dilator muscle [8, 9]. The relative tone of the iris sphincter and dilator muscles determines pupil diameter. These mechanisms are generally well understood within the context of the pupil light reflex (PLR), which elicits very large changes in pupil diameter (sometimes exceeding 100% of baseline diameter) [10]. The task-evoked pupil response, however, is much smaller in magnitude (increasing up to about 10–20% from baseline [2]), and less well understood. When the pupil dilates in response to changing mental states, it is thought that an inhibitory mechanism is acting upon the parasympathetic constriction pathway, via modulation of the LC-norepinephrine system [2, 11].

The LC is a subcortical brain structure that is responsible for the modulation of norepinephrine (NE) release, with dense innervation to brain regions involved in selective attention. Current models of selective attention and attention networks in general include critical roles of the LC and NE [12–14], and studies in both animal models and humans show tight links between activation of the LC-NE system and pupil dilation responses [15–17]. The adaptive gain theory (AGT), proposed by Aston-Jones and Cohen, posits that attentional control states are continually modulated by tonic and phasic neural activity in the LC, and this modulation can be reflected in shifts of tonic and phasic pupil diameter [15, 18]. Tonic pupil diameter considers the relatively sustained component of pupil response, is typically calculated by averaging a relatively prolonged duration of basal or task-engaged pupil diameter, and is generally modulated by arousal level, lighting conditions, and cognitive workload [19]. In contrast, phasic pupil diameter considers the relatively transient and event-related component of pupil response (i.e., the task evoked pupillary response [20]), is typically calculated relative to the onset or offset of an event (e.g., a stimulus appearance), and is generally modulated by task difficulty, violation of expectations, and uncertainty of outcomes [21].

The AGT model distinguishes between two specific attentional control states: exploration and exploitation. The exploration state is characterized by tonic LC activity associated with diffuse attention and sensitivity to salient stimuli. The exploitation state is characterized by phasic LC activity that signals a shift to more focused and exploitative attention. These two states are reflected in pupil diameter. For example, tonic pupil diameter is generally higher as difficulty increases on a task [22, 23] and increases immediately before a participant shifts from an exploitation to an exploration state; in general, this pattern is thought to indicate higher tonic LC firing rates during more challenging tasks. In contrast, shifting from an exploration to exploitation state is associated with sudden phasic dilatory responses [24], as seen when highly task-relevant stimuli are presented; in general, this pattern is thought to reflect phasic firing rates in the LC. The second pattern of results is also predicted by recent theory proposing that task-evoked pupil dilation indicates an update of an emergent mental representation [25]; specifically, as the brain detects divergence between prior and posterior representations, the pupil shows phasic dilation.

Most tests of these theories with humans are done with highly controlled cognitive tasks. For example, listening for rare (oddball) sinusoidal tones [26], or betting on payoffs during a highly structured, simulated gambling task [27]. The present study examined whether pupil diameter might indicate exploration-exploitation dynamics while physicians continuously search, interpret, and diagnose digital whole slide images (WSI) of breast biopsies that vary in representation of disease progression and diagnostic difficulty. When pathologists view digital WSIs, their goal is to search the image for suspicious lesions, recognize critical histopathological features, and then map those features onto an appropriate diagnostic category (e.g., benign tissue versus potential cancer) [28–32]. In doing so, they generally search the scene at low magnification and then zoom to high magnification to interrogate specific features [29, 31, 33]. We propose that pathologists are constantly choosing between continuing to search for and gather information (i.e., explore at low magnification) versus deeming current knowledge sufficient to decide upon a diagnostic category (i.e., exploit at high magnification). This bears some similarity to explore-exploit trade-offs seen in reinforcement learning: participants must choose between continuing to explore (low value) areas in an attempt to find rewarding (high value) information that will maximize rewards [34, 35]. In pathology, the naturalistic shifts from low to high magnification (power) provide opportunities for testing predictions of the AGT in a relatively real-world context. According to existing research, pupil diameter during exploratory search (i.e., at low magnification) should be associated with image difficulty, indicating adaptive tonic LC-NE tone. Also, phasic pupil diameter should reflect shifts from exploratory to exploitative modes (and/or vice-versa), indicating adaptive phasic LC-NE tone.

We investigate four primary hypotheses. First, during low-magnification search (e.g., 1× to 5× zoom), we will find larger tonic pupil diameter when pathologists examine images with relatively high standardized difficulty ratings; such a pattern would suggest relatively high tonic LC-NE activity during exploration of more challenging stimuli. Second, we expect that tonic pupil diameter will be associated with zoom level, with higher zoom levels (i.e., exploitation) generally associated with lower pupil diameter. Third, we hypothesize that immediately prior to and during a zoom-in event from low- to high-magnification (e.g., shifting from 1× to 10× zoom), we will find a pronounced phasic pupil dilation; such a pattern would suggest that zoom-in events are associated with high phasic LC-NE activity and a shift from exploratory to exploitative behavior. Finally, we hypothesize that *before* a zoom-out shift from exploitation (high magnification) to exploration (low magnification), pupil diameter will be higher. These four hypotheses are summarized in Table 1.

**Table 1:** Study hypotheses.

Hypothesis Number	Analysis Type	Conditions	Zoom Level	Expected Pattern	Clinical Rationale
1	Tonic	Low vs. High Difficulty Images	Low magnification (1× to 5×): exploration	Larger pupil diameter during more difficult interpretation.	More visually or cognitively demanding feature interpretation.
2	Tonic	Low vs. High Magnification Zoom	Low (1× to 5×) and High (6× to 60×) magnification	Larger pupil diameter during exploration (low magnification) vs. exploitation (high magnification).	Low magnification visual scan of image to find regions to interrogate.
3	Phasic	All Images	Shift from low to high magnification: shift to exploitation	Pupil diameter increase upon zoom-in event.	Shift to high magnification to interrogate concerning features.
4	Phasic	All Images	Shift from high to low magnification: shift to exploration	Pupil diameter is higher before zoom-out event.	Features interpreted, ready to shift back to low magnification scanning.

The four primary hypotheses of our study, clinical rationale for the hypotheses, and how the hypotheses were tested.

## Materials and methods

### Participants

To calculate a sample size estimate we used mean effect sizes (Cohen’s D = 0.55) reported in a study examining task-evoked pupil diameter while participants interpreted visual stimuli of varied difficulty [36]. With α = 0.05 and power = 0.95, a minimum sample size of 38 participants is advised. To increase our statistical power, we collected data from 89 pathologists as part of a larger study examining how resident physicians’ diagnostic expertise and eye movements change through their specialized training.

To reduce sampling bias, we intentionally recruited a geographically and experientially diverse sample of pathologists. This included recruiting participants from nine major university medical centers distributed across the United States (in eastern, western, northern, and southern regions of the country); the participants held highly varied experience levels, including 70 resident pathologists and 19 experienced (faculty) pathologists. While we cannot control which pathologists chose to participate in our study or guarantee that our results will generalize to other groups of pathologists or other specialized domains of medicine, we are confident that our recruitment and data collection procedures reduced selection bias. Sample characteristics are detailed in Table 2.

**Table 2:** Demographic information.

Participant Group	Variable	Data
**Trainees in Pathology Residency or Fellowship Program**	Year of Training in Pathology	Year 1 Resident: N = 19
		Year 2 Resident: N = 25
		Year 3 Resident: N = 18
		Year 4 Resident: N = 7
		Fellow: N = 1
	Approximate Weeks of Breast Pathology Training	Year 1 Resident: *M* = 3.24
		Year 2 Resident: *M* = 5.5
		Year 3 Resident: *M* = 7.47
		Year 4 Resident: *M* = 9.29
		Fellow: *M* = 17.0
	Sex	Male: N = 34
		Female, Other, or Undisclosed: N = 36
**Experienced Pathologists**	Total Years of Experience Interpreting Breast Pathology	<1–4 years: N = 4
		5–9 years: N = 5
		10–19 years: N = 7
		20+ years: N = 3
	Percentage of Breast Cases in Current Case Load	< 10%: N = 3
		10–24%: N = 3
		25–49%: N = 4
		50–74%: N = 7
		75% or more: N = 2
	Fellowship Trained in Breast Pathology?	Yes: N = 7
		No: N = 12
	Sex	Male: N = 8
		Female, Other, or Undisclosed: N = 11

Demographic details for the participating pathologists (N = 89).

All participants provided written informed consent, and all study procedures were approved by the appropriate Institutional Review Boards (IRB), with the University of California, Los Angeles acting as the IRB of record (Protocol #18–000327).

### Materials

A pool of 32 high resolution, zoomable (1× to 60×), breast tissue biopsy images (digital whole slide images) were used in this study; each image represented a single patient’s breast biopsy and was selected from a larger set of standardized images developed in earlier research [37, 38]. A panel of expert pathologists determined a single consensus reference diagnosis for each image [37, 39], and images were selected to represent the full spectrum of diagnoses, ranging from benign to invasive cancer. Specifically, there were 4 images defined by the consensus as benign breast tissue without atypia, 10 as atypia, 10 as low-grade ductal carcinoma in situ (DCIS), 4 as high-grade DCIS, and 5 as invasive carcinoma. In general, progression along this continuum from benign to invasive carcinoma is associated with more aggressive clinical treatment and surveillance. For a description of low- versus high-grade DCIS, see prior work [40].

The selected images also varied in standardized difficulty ratings. In general, images representing the polar ends of the diagnostic continuum, benign and invasive, are less challenging to diagnose and show higher agreement among practicing pathologists [39]. More challenging, however, are images representing the atypia and DCIS categories. For our selected images, standardized difficulty ratings for each of the 32 study images were derived from a separate group of 54 experienced breast pathologists who rated image difficulty on a scale from 1 (very easy) to 6 (very challenging) (Mean = 3.0, Median = 3.0, StDev = 0.7) [39]. All participants began with a practice image; an invasive carcinoma image that elicited very low difficulty ratings (*M* = 1.71) and high diagnostic accuracy (*M* = 0.78) in the earlier participant sample.

For the purposes of this paper, accuracy is defined as concordance with the consensus reference diagnosis defined by an expert panel (as previously detailed [38]); for example, if a participating pathologist diagnosed an image as Invasive, and the expert consensus reference diagnosis is Invasive, that is scored as accurate. Any deviation from this concordance, to either a higher category (e.g., consensus Atypia, participant diagnosed as Invasive) or a lower category (e.g., consensus Invasive, participant diagnosed as Atypia), was scored as inaccurate. Because there are five diagnostic categories, an observer who randomly and uniformly assigned a diagnosis would average 20% accuracy.

The non-practice images were divided into three test sets of 14 images, each containing two benign images, four atypia images, four low-grade DCIS images, two high-grade DCIS images, and two invasive images. As described in the Data Collection Locations and Procedures section, each participating pathologist interpreted one set of 14 images.

### Equipment

We used a Dell Precision M4800 laptop and a SensoMotoric Instruments (Boston, MA) Remote Eye Tracking Device (RED; 250Hz, ≤ 0.5˚ gaze position accuracy) attached to a 22” color-calibrated Dell liquid crystal display (LCD) monitor (at 1920 × 1080 resolution). Images were displayed on a custom digital viewer software using the DeepZoom Silverlight application (Microsoft, Inc.) in the web browser. The viewer tool allowed participating pathologists to zoom (1× to 60×) and pan the digital whole slide images while maintaining high resolution, and make measurements and annotations. While a pathologist used the viewer tool, data were automatically logged (at approximately 10Hz) to a local SQL database, to include zoom, position, and annotation data.

To collect diagnostic information from participants after reviewing each image, we developed a histology form on the Qualtrics web-based platform; histology forms are commonly used by pathologists to record their observations and interpretations of images [31, 41]. The histology form asked participating pathologists to provide the single most advanced diagnosis by selecting among the five diagnostic categories detailed above (ranging from benign to invasive carcinoma). They also rated the difficulty of interpreting the image and their confidence with their determination (both on scales from 1–7). Of less relevance to this paper, information was also gathered about histopathological features (e.g., nuclear grading, presence and nature of necrosis, mitotic activity), whether they believed the features indicated a borderline diagnosis (between two diagnostic categories), and whether the pathologist would seek a consultative second opinion if they encountered the image during ordinary clinical practice.

### Data collection locations & procedures

Following informed consent and prior to reviewing the images, participating pathologists completed a baseline survey probing basic demographic information (results in Table 2).

**Fig 1:**
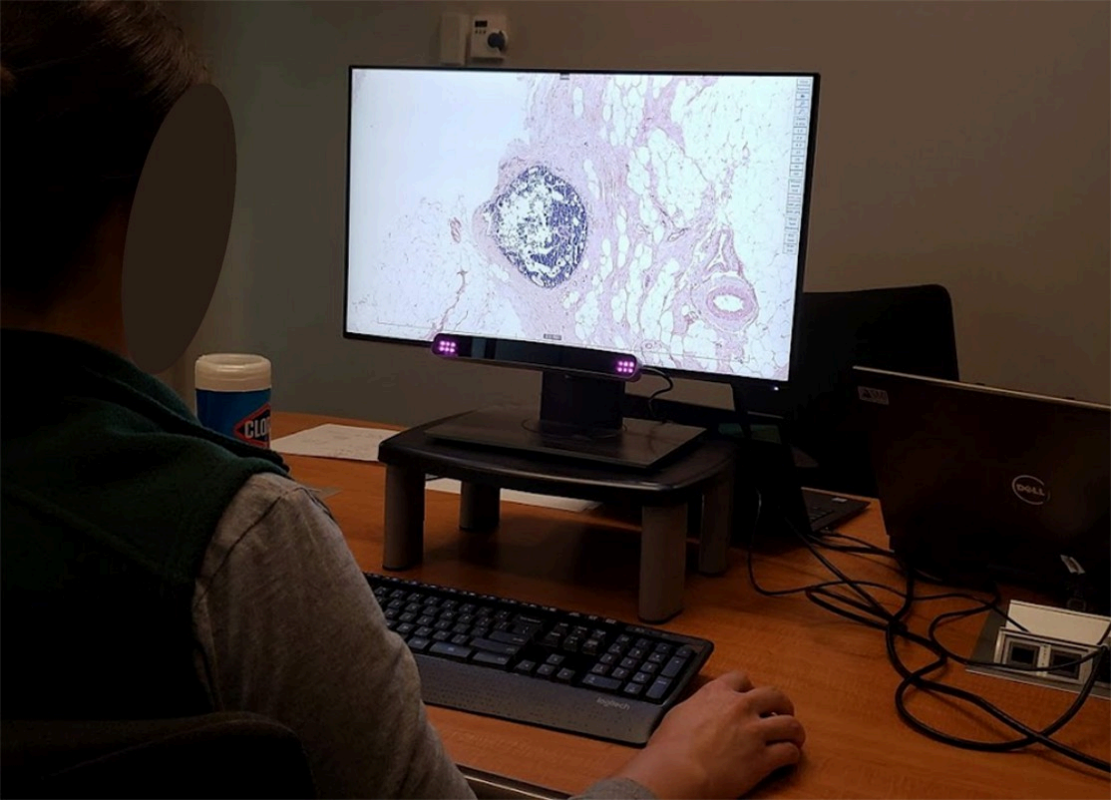
Experimental apparatus. A participating pathologist (face obscured for privacy) interpreting a breast biopsy image during the study, navigating the image by using the computer mouse to click-drag (panning in XY space) and scroll (zooming in Z space). The remote eye tracking device is attached to the bottom of the computer monitor.

The investigators (TTB, TD) traveled to each of the nine data collection sites, bringing one of two system setups (computer, software, eye tracker) matching the specifications described in the Equipment section. At each site, data collection was completed in a private office or conference room, examining one participant at a time for an approximately one-hour session. Following a nine-point eye tracker calibration, participants practiced using the user interface (zooming, panning, drawing regions of interest) while briefly interpreting the practice image; they also practiced completing the histology form and had the opportunity to ask the experimenter any questions. Following the practice phase, this same process (view and interpret the image, complete the histology form) was completed for each of the 14 experimental images (drawn from the pool of 32 images described in the Materials section. After interpreting the 14 images, participants were compensated with a $50 USD gift card. Fig 1 depicts the experimental apparatus.

### Data scoring

Two independent data streams were logged during the pathologists’ interpretation of images: 1) a series of position information in coordinate space (XYZ) over time reflecting navigation through the image output by the viewer software and stored in a local SQL database, and 2) the eye tracker’s data output (at 250Hz) reflecting gaze position and pupil diameter over time. These two data streams were merged into a single file by leveraging common system time stamps, allowing us to co-register pupil diameter changes (recorded by the eye tracker) with changes in zoom level (recorded by the viewer tool). As in prior related work, artifacts and missing data due to blinks were remedied with linear interpolation, and we focused our analysis exclusively on right eye pupil diameter [24, 42, 43].

To assess tonic pupil diameter and enable testing of hypotheses 1 and 2, we assessed ongoing pupil diameter during the entire image viewing period, calculating mean pupil diameter in millimeters.

To assess phasic pupil diameter prior to, during, and following zoom events (i.e., to enable testing of hypotheses 3 and 4), we needed to identify discrete zoom-in and zoom-out events in the continuous data stream. To do so, we examined the continuous zoom data and noted instances of successive increases or decreases in magnification over time. The first magnification change in a sequence (e.g., from 1× to 2×) was marked as the beginning of a sequence, and the last was marked as the end of a sequence. Using this method, a total of 21,416 zoom events were identified, 11,326 involved increases in magnification (i.e., zoom-in) and 10,090 involved decreases in magnification (i.e., zoom-out).

These identified zoom events varied dramatically in duration, amplitude, and latency relative to adjacent zoom events. To ensure that each zoom event was relatively discrete (i.e., brief in duration), pronounced (i.e., high in amplitude), and independent (i.e., relatively latent), we developed three criteria for the inclusion of zoom events in analysis.

First, we set a criterion of a 5 second maximum zoom event duration. Examining all 21,416 zoom events, duration ranged from 100 ms to 20.7 s, with a very strong positive skew (Fisher’s skewness = 2.42). The right tail of the distribution was particularly extended after 5 seconds duration, extending out to 20.7 seconds but only comprising about 7% of the distribution. Because we desired to only include zoom events that were relatively discrete rather than extended and continuous (e.g., gradually increasing zoom in a stepwise manner over the course of 20 seconds), we chose to use a 5 second maximum zoom event duration, allowing us to retain 92.8% of all zoom events. If we had included relatively lengthy zoom events in our analyses, these epochs would be more likely to include the viewing of highly disparate image regions, at different magnification levels, and with multiple discrete magnification changes; we believe these confounds would have reduced the interpretability of results. Furthermore, our decision to exclude relatively lengthy zoom events is congruent with most analyses of phasic pupil diameter, which typically consider a 3–5 second pupil response to a discrete and brief event (e.g., ≤ 5-second presentation) [24, 26, 44–46].

Second, we set a criterion of a 5× minimum zoom event amplitude. Examining all 21,416 zoom events, amplitude ranged from a minimum magnification change of 1× to a maximum of 59×, with a positive skew (Fisher’s skewness = 1.81). The left tail of the distribution was characterized by many very low amplitude changes, for example a zoom change from 3× to 4× magnification. Because we desired to only include zoom events that were relatively pronounced and related to a meaningful change in informational content in the image, we chose to only include zoom events with a 5× minimum zoom change amplitude. This criterion allowed us to retain 66.4% of all zoom events. In discussions with experienced pathologists, it was determined that relatively small (e.g., 1-4x) changes in zoom level do not typically change the information available to the pathologist; in other words, very small zoom changes are unlikely to cause additional histopathological features to become perceptible. Rather, the visual features available for inspection typically change (from relatively architectural to cellular) with more pronounced magnification changes between 5-20x. Because we only desired to include zoom events that were relatively discrete, pronounced, and associated with meaningful changes in information available to the participant, we believe that excluding very minor zoom changes was an important step in our data processing.

Third, latency was the biggest challenge we dealt with during data processing, resulting in the most pronounced removal of data. Because prior research [26] demonstrates that pupil diameter data can be “confounded by pupil diameters on trial *n*– 1” (p. 255), we wanted to ensure there was no overlap of pupil data within 5 seconds before or after the onset of a zoom event. Latency between adjacent zoom events ranged from a minimum of 196 ms to a maximum of 136.4 s, with a very strong positive skew (Fisher’s skewness = 4.76). Most zoom events (90.4% of all zoom events) occurred within 5 seconds of another zoom event, leaving only 9.6% of all zoom events for analysis. While this criterion causes a dramatic reduction of data to be analyzed, we believe it is necessary to avoid overlapping pupil data between zoom events, which would render our results difficult or impossible to interpret. Specifically, early event-related pupil diameter waveforms could be contaminated by pupil responses continuing from an immediately preceding event.

Together, our three criteria allowed us to retain 1,139 zoom events for analysis, including 672 zoom-in and 467 zoom-out sequences, distributed across 87 of the 89 participants (resulting in two participants being removed). Analyzing 1,139 critical zoom events provides high power to detect differences between task conditions; indeed this number of trials is similar to the number analyzed in prior studies examining pupil responses during decision making tasks (e.g., 1000 trials [47], 1065 trials [48], 1200 trials [49]).

### Data analysis

It is possible that there would be different ratios of tissue to background (white) space when participants move between zoom levels. Biopsy tissue tends to have dark blue to purple and pink regions (resulting from standard hematoxylin and eosin staining) superimposed against a white background. As a pathologist zooms in, more of the screen might be occupied by relatively dark tissue regions, causing increased pupil diameters due to lower luminance and thus systematic differences in pupil diameter as a function of zoom level.

To measure image luminance, we used the MATLAB (Mathworks, Inc., Natick, MA, USA) Image Processing Toolbox to analyze luminance level of the area (20˚) surrounding participant’s eye fixations on the 32 images at each zoom increment. We used mean luminance value (measured in V, of HSV) as a covariate in our analyses of tonic and phasic pupil diameter.

Analysis proceeded through four phases, one for each hypothesis. First, we tested Hypothesis 1: that tonic pupil diameter would be larger during more difficult image interpretations while pathologists explored at low magnification. To do so, we identified low magnification (1× to 5×) epochs during image review, then calculated mean tonic pupil diameter during those epochs, excluding all periods when zoom level was >5× (77% of all data). This provided a single, mean pupil diameter data point for each participant and each image. Using SPSS v21 (IBM, Inc., Armonk, NY), we then fit a generalized estimating equation (GEE) model with pupil diameter as the quantitative outcome, including image ID as fixed effects, and the rated difficulty of each image as the explanatory variable of interest. Mean luminance was included as a covariate, and because each participant reviewed 14 images, we treated each participant as a cluster in the GEE model.

Second, we tested Hypothesis 2, that tonic pupil diameter would be larger during exploration (low magnification zoom) than exploitation (high magnification zoom). To do so, we calculated mean pupil diameter at each zoom level (1× to 60×) for the entire duration of an image review. This provided up to 60 mean pupil diameter data points (*M* = 20.04 data points) for each participant and each image; note that in most cases there were far fewer than 60 because most participants never achieved 60× zoom. We then fit a GEE model with pupil diameter as the quantitative outcome, including image ID as fixed effects, luminance as a covariate, and zoom level as the explanatory variable of interest (and each participant as a cluster).

Third, we tested Hypothesis 3, that shifts in zoom from low- to high-magnification would be associated with larger pupil diameter. To do so, we plotted mean phasic pupil diameter for the 5 seconds prior to (i.e., -5000ms) and following (i.e., 5000ms) a zoom-in event, in 4ms increments, zero-referencing mean pupil diameter to 5000ms prior to the onset of the zoom event (i.e., time -5000). We calculated mean pupil diameter for the two seconds immediately before (from -4 to -2004ms) and after (from 4 to 2004ms) the zoom-in event. We fit a GEE model with a fixed effect for each participant, asking whether time (pre vs post zoom-in) was associated with phasic pupil diameter. We also conducted exploratory analyses to examine whether the magnitude of the phasic zoom-in pupil response was modulated by participant- or image-level variables. To do so, we analyzed the signed peak amplitude of the phasic pupil response for each zoom-in event, focusing on the time immediately following the onset of the zoom (i.e., 0 to 2000ms). We used this quantitative measure as the outcome in a series of GEE models, including image ID as fixed effects, and several exploratory variables of interest (with participant always included as a cluster).

Finally, we tested Hypothesis 4, that pupil diameter would increase before a high- to low-magnification zoom event. To do so, we compared mean pupil diameter for each participant and image during two time periods preceding a zoom-out event: one immediately prior to the zoom-out event (i.e., -1000 to 0ms), and one relatively latent time window (i.e., -2004 to -1004ms). We fit a GEE model with a fixed effect for each participant, asking whether time (latent versus immediately prior to a zoom-out event) was associated with phasic pupil diameter. We also conducted exploratory analyses to examine whether the signed peak amplitude of the pupil response immediately prior to the zoom-out event (i.e., 0 to -2000ms) was modified by participant- or image-level variables.

For each analysis, significant statistical test results are provided as Wald’s chi-square (χ^2^) along with β estimates, standard error (SE), and *p*-values. Non-significant results are included in Table 3.

**Table 3:** Statistical test results.

Hypothesis	Statistical Test	Wald χ^2^	p-value
1	Interaction: Experience Level × Rated Difficulty	0.19	0.66
	Interaction: Accuracy × Rated Difficulty	0.82	0.37
2	Interaction: Rated Difficulty × Zoom	0.20	0.65
	Interaction: Experience Level × Zoom	< 0.01	0.97
	Interaction: Accuracy × Zoom	0.43	0.51
3	Main Effect: Rated Difficulty	< 0.01	0.98
	Main Effect: Zoom-in Magnitude	0.57	0.45
	Main Effect: Experience Level	0.17	0.68
	Main Effect: Accuracy	0.57	0.45
4	Main Effect: Rated Difficulty	0.04	0.85
	Main Effect: Zoom-out Magnitude	<0.01	0.99
	Main Effect: Experience Level	2.44	0.12
	Main Effect: Accuracy	0.92	0.34

Statistical test results for non-significant effects, separated by hypothesis.

After testing our primary hypotheses of interest, we also conducted separate follow-up analyses to explore additional questions that were not guided by hypotheses, but we anticipated could be of interest. We conducted separate analyses to answer these secondary/exploratory questions so as not to distract from the primary aims of this investigation, and because questions concerning possible effect modification would necessarily have lower power compared to analyses of main effects. Furthermore, including effect modification terms in our primary analyses would render the R values, coefficients, and p-values associated with our primary outcomes difficult to interpret in a meaningful way. For these reasons, our follow-up analyses stand alone.

## Results

### Hypothesis 1

**Fig 2:**
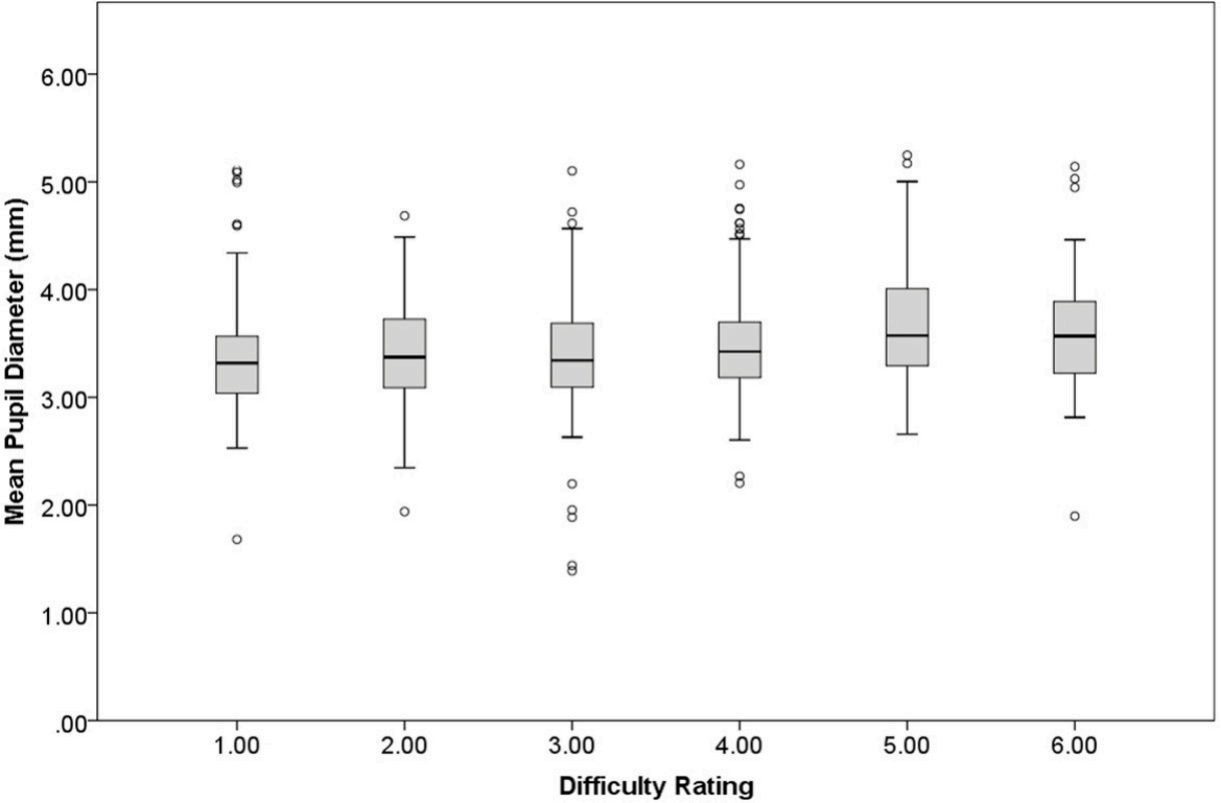
Hypothesis 1 tonic pupil diameter. Mean tonic pupil diameter (in mm) as a function of pathologists’ difficulty rating of each image. Boxplot represents data median (horizontal line), quartiles (shaded), minimum and maximum (error bars), and outliers (dots: 1.5 times the interquartile range).

We found a small but statistically significant association between difficulty level and tonic pupil diameter, χ^2^ = 7.47, β = 0.05, SE = 0.02, *p* < .01 (Fig 2). Higher-rated difficulty was associated with larger tonic pupil diameters while participants explored the images at relatively low magnification (i.e., 1× to 5×), even when accounting for screen luminance, supporting Hypothesis 1.

We conducted follow-up analyses to explore whether the association between difficulty level and tonic pupil diameter varies by pathologists’ training status (1 = resident, 2 = faculty), or by the final diagnosis given to the image by the participant. To do so, we entered difficulty level and one of those two variables as explanatory variables of interest (along with luminance as a covariate) in a series of two GEEs models, testing for two-way interactions. There were no significant interactions (Table 3).

A final follow-up analysis explored whether the association between difficulty level and tonic pupil diameter was specific to low-magnification zoom, or if an association exists during periods of time characterized by high-magnification zoom (i.e., 6× to 60×). This analysis showed a very similar pattern to our first analysis, with difficulty level associated with tonic pupil diameter, χ^2^(1) = 6.92, β = 0.05, SE = 0.02, *p* < .01. Specifically, higher rated difficulty was positively associated with larger tonic pupil diameters while participants were at relatively high magnification (i.e., ≥ 6× zoom). Notably, the strength of the association between difficulty and pupil diameter is very similar across low versus high zoom levels.

### Hypothesis 2

**Fig 3:**
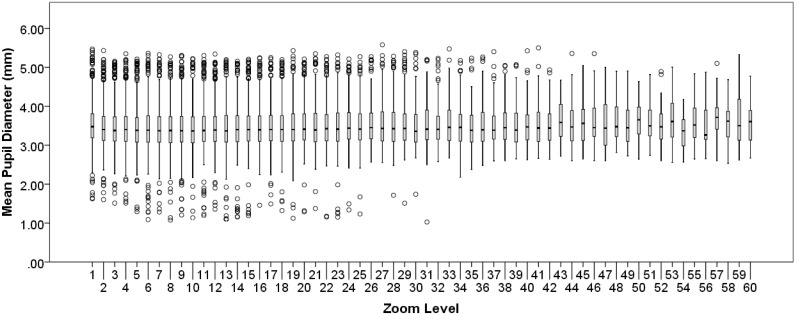
Hypothesis 2 tonic pupil diameter. Mean tonic pupil diameter (in mm) as a function of zoom level. Boxplot represents data median (horizontal line), quartiles (shaded), minimum and maximum (error bars), and outliers (dots: 1.5 times the interquartile range).

On average, 1 higher Zoom level was associated with 0.01 larger tonic pupil diameter, χ^2^(1) = 6.19, β = 0.01, SE < 0.01, *p* = .013 (Fig 3). The observed association is opposite the negative association predicted by Hypothesis 2.

We conducted three follow-up analyses to explore whether the association between zoom level and tonic pupil diameter might be modified by each pathologist’s difficulty rating of each image, training status (1 = resident, 2 = faculty), or the accuracy of the final diagnosis. To do so, we entered zoom level and one of those variables as explanatory variables of interest (along with luminance as a covariate) in a series of three GEEs, testing for two-way interactions. There were no significant interactions between zoom level and any of the three variables.

### Hypothesis 3

**Fig 4:**
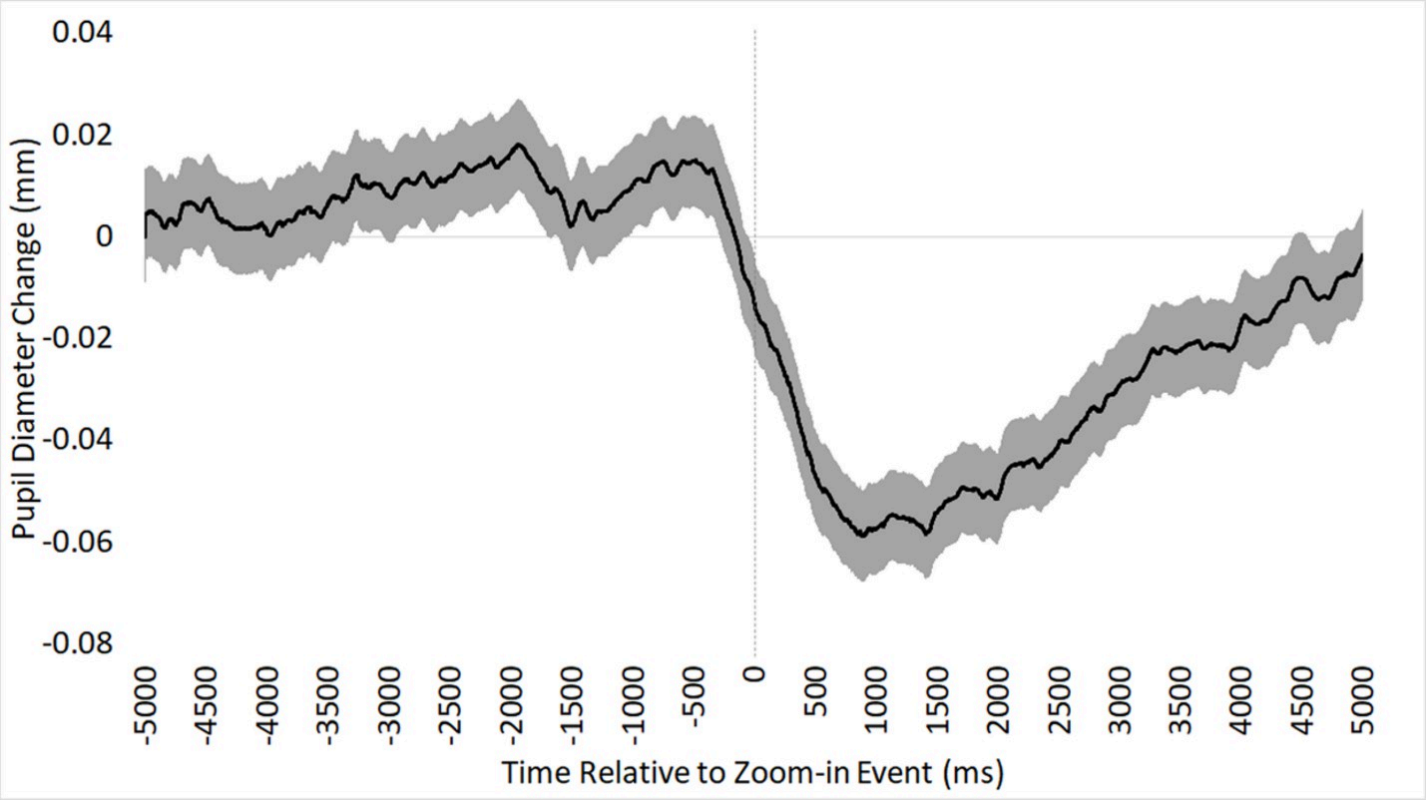
Hypothesis 3 phasic pupil diameter. Mean phasic pupil diameter response to zoom-in events over time (in ms), time-locked to the onset of a zoom-in event. Shaded region indicates standard error of the mean.

On average, pupil diameter was 0.053mm higher before (*M* = 0.008) versus after (*M* = -0.045) a zoom-in event, χ^2^(1) = 38.08, β = 0.053, SE < 0.01, *p* < .001, as depicted in Fig 4. This pattern is the opposite of what we predicted in Hypothesis 3.

We built four exploratory follow-up GEE models to examine possible associations with the amplitude of the phasic pupil change in response to zoom-in events on each image. Each model included one of the following four explanatory variables of interest (along with mean post-event luminance as a covariate): the difficulty rating of each image, the magnitude of the zoom-in event, pathologists’ training status (1 = resident, 2 = faculty), and the accuracy of the final diagnosis. No variable of interest was significantly associated with the amplitude of the phasic pupil diameter change (Table 3).

### Hypothesis 4

**Fig 5:**
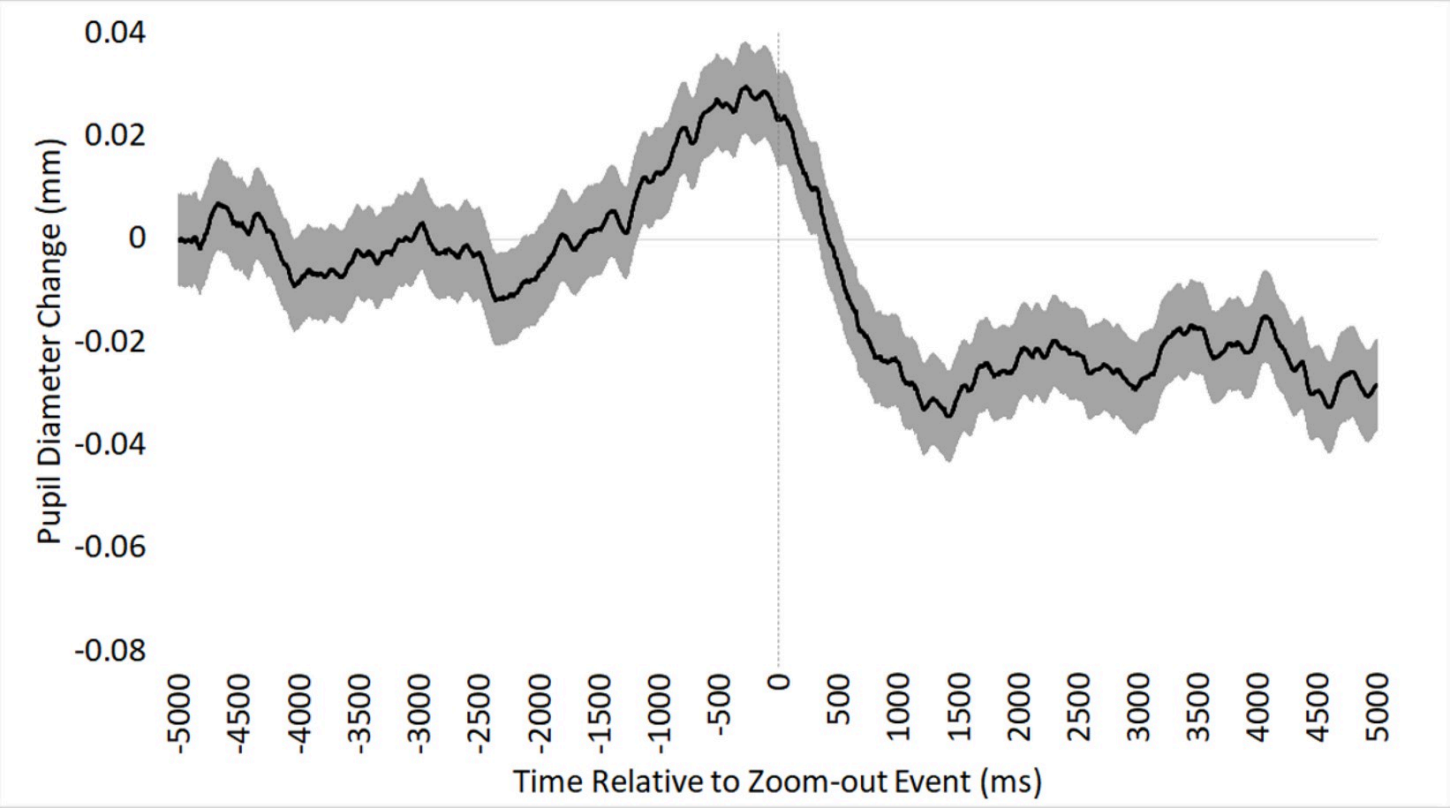
Hypothesis 4 phasic pupil diameter. Mean phasic pupil diameter response to zoom-out events over time (in ms), time-locked to the onset of a zoom-out event. Shaded region indicates standard error of the mean.

On average, pupil diameter was 0.02mm higher immediately prior to (*M* = 0.007) versus relatively latent to (*M* = -0.013) a zoom-out event, χ^2^(1) = 5.43, β = 0.02, SE < 0.01, *p* < .05, as depicted in Fig 5. Specifically, pupil diameter increased immediately prior to a zoom-out event, supporting Hypothesis 4.

We built four exploratory follow-up GEE models to examine possible associations with the magnitude of each participant’s phasic pupil change immediately preceding the zoom-out event. Each model included (pre-event) luminance as a covariate, along with one of the following four explanatory variables of interest: the difficulty rating of each image, the magnitude of the zoom-out event, pathologists’ training status (1 = resident, 2 = faculty), and the accuracy of the final diagnosis. None of the variables of interest was significantly associated with the magnitude of the pupil diameter increase prior to a zoom-out event (Table 3).

## Discussion

The present study investigated whether relatively naturalistic pupil diameter changes during the continuous review of digital WSIs would support four predictions derived from AGT. We found mixed support for our hypotheses.

### Theoretical implications

First, we expected that tonic pupil diameter during low-magnification visual search would increase as perceived difficulty of the task increased. This hypothesis was supported. Specifically, an increment on the 1–7 difficulty rating scale was associated with a pupil diameter increase of approximately 0.05mm. This translates to an approximate pupil diameter change of 0.35mm across the scale, which is approximately a 10% increase, similar to what is found in studies manipulating task difficulty [4, 5, 25, 50–52]. We found a very similar pattern when examining tonic pupil diameter during the exploration of images at relatively high zoom levels. There are at least three possible explanations for increases in tonic pupil diameter during relatively difficult image review. First, according to the AGT, increased difficulty is often associated with increased expected value of a task, which can lead to increased tonic pupil diameter during exploration of more challenging images [26]. Second, Information Gain theory proposes that pupil diameter increases under conditions of relatively high uncertainty due to the amount of mental effort, including memory retrieval demands and increasing load on working memory, to process information and accumulate sufficient evidence to resolve uncertainty [25]. A fuller description of Information Gain theory in the context of diagnostic pathology would involve an assessment of possible disease states and their respective probabilities, how information available from various histopathological features maps to possible interpretations, how noise corrupts this mapping and introduces uncertainty, and how this influences diagnostic interpretation [53]; while outside the scope of this study, quantifying these information-theoretic variables remains an exciting direction for future research. Finally, Attentional Learning Theory suggests that pupil dilation under more difficult conditions may be linked to visual cues that caused high prediction error in the past [54, 55]. Trained pathologists may recognize specific visual features of images that caused them to be led astray in the past, causing pupil dilation that may reflect an error-driven attentional process.

Our second hypothesis proposed that tonic pupil diameter would decrease as a function of increasing zoom level, under the assumption that higher zoom levels are more likely to be associated with exploitation and smaller pupil diameters, relative to exploration. Interestingly, we found the opposite pattern: tonic pupil diameter tended to be larger with higher zoom level. While higher zoom levels are also associated with higher screen luminance, we accounted for the potential confounding effects of luminance by including it as a covariate in our analytic model. The fact that pupil diameters were larger at higher zoom levels could be attributed to higher zoom levels being associated with more challenging tasks for the pathologist. At higher zoom levels, pathologists are tasked with examining low-level histopathological features at the cellular level that, in some cases, may be challenging to interpret or at least involve higher load on attention, working memory, or memory retrieval processes. In other words, larger tonic pupil diameter at higher zoom level may be a byproduct of one fundamental aspect of digital WSI: the deeper you zoom, the more (and potentially more challenging) information is available for inspection. This is the only putative explanation we can identify for the unexpected findings. However, if this were the case, one might expect the relationship between zoom level and tonic pupil diameter to be modulated by the rated difficulty level of the image; for example, more difficult images should be associated with more increased pupil diameters at higher zoom level, but that analysis yielded a non-significant interaction. Of course, the difficulty of specific encountered features over time may or may not be related to an overall difficulty rating of the image, which may better reflect difficulty mapping identified features to candidate diagnoses. Unfortunately, while we have no method for associating specific visual features with their inherent difficulty, this remains an open direction for future research to disentangle the relatively transient (i.e., feature-related) versus enduring (i.e., image-related) difficulty experienced during image interpretation.

Our third hypothesis concerned zoom-in events, where we expected to see a shift from exploration to exploitation as indicated by phasic pupil dilation. Interestingly, we found the opposite pattern: when participants zoomed in, they showed a pronounced phasic pupil constriction of about 0.053mm. This phasic response tended to begin about a half second prior to the onset of the zoom event, and then return to pre-zoom levels after about 5 seconds (Fig 4). This result was unexpected and difficult to interpret, but it could reflect the pupil’s response to the onset of motion during a zoom.

Our fourth and final hypothesis concerned zoom-out events, where we expected to see a phasic pupil diameter increase immediately prior to the shift from exploitation to exploration. This hypothesis was supported by our data, which showed an approximately 0.02mm phasic dilatory response immediately prior to the onset of a zoom-out event. We believe this overall pattern reflects a resolution of evidence accumulation during a high-magnification exploitation of visual features, providing pathologists with sufficient confidence to return to low magnification and either terminate their search (and proceed to the diagnostic histology form) or continue their search in a new location. In some cases, resolution of evidence accumulation can be a cyclical process for a pathologist; for example, repeatedly zooming in to exploit information at high magnification, perhaps to rule out alternative diagnostic hypotheses [56, 57]. While we did not find any significant evidence of an association between the magnitude of zoom-out events and diagnostic accuracy, prior research shows that low-magnification scanning of an image (i.e., less zooming-in) is associated with higher accuracy [31], and repeatedly zooming-in on different areas of the image can lead to over-interpretation of features and misdiagnosis [30]. Another potential interpretation of the pupil dilatory response immediately prior to a zoom-out event is pathologists doing any last-second confirmatory checks that nothing in the current view was missed (or misinterpreted), prior to shifting tasks. Overall, we view our data as supporting our fourth hypothesis, demonstrating an association between zoom-out behaviors and adaptive shifts of control states (i.e., exploitation to exploration).

During both zoom-in (hypothesis 3) and zoom-out (hypothesis 4) events, we found similarly pronounced phasic pupil constriction around the onset of the zoom event. While it is unclear why this occurred, it could be associated with perceptual aspects of the zoom event itself, rather than a reflection of shifting control states. For example, zooming in or out may cause brief blurring of the scene, a temporary loss of spatial localization within the image, the revealing of new structures and colors, and the perception of optic flow. Indeed, past research has found that the pupils tend to constrict in response to perceiving changes in visual structure, color, or motion [58–61]. It could be the case that the visual characteristics of a zoom event are causing the pronounced pupil constriction showcased in Figs 4 and 5. However, this possibility is difficult to reconcile with our finding that the magnitude of a zoom-in event was not associated with the magnitude of the pupil constriction. Continuing research may find value in experimental attempts to disentangle pupillary responses to changes in visual features versus changes in control states.

### Practical implications

As pathologists interpret and diagnose WSIs, they must search for critical regions of a visual scene where informative features are likely to be found, recognize important features that are relevant to the task, and then successfully interpret the features and map them to a final diagnosis [32, 62–64]. Each phase of this interpretive process is vulnerable to errors [32, 62, 63, 65], providing opportunities for eye tracking to provide novel insights into the conditions under which errors emerge, and using these insights to inform medical education, training, and competency assessment [66]. For example, eye tracking may prove valuable for automated competency assessments, allowing instructors to gain novel insights into when students are failing to find, examine, assign value to, or correctly interpret critical regions. Alongside fixation-related eye data, tonic and phasic pupil diameter could help elucidate when pathologists sufficiently recognize the inherent difficulty of an image interpretation, and when they realize the relevance or ambiguity of critical regions.

Regarding tonic pupil diameter changes, the present results add to a growing body of evidence that larger tonic pupil diameter is generally associated with higher difficulty ratings of digital WSIs [22, 41]. For students, the ability to effectively gauge the difficulty of encountered features is a hallmark ability of gaining expertise in a domain [67, 68]. By using WSIs with standardized difficulty ratings achieved through consensus, pupil diameter could provide a novel quantitative metric for assessing whether students recognize the inherent difficulty or complexity of a novel diagnostic challenge.

Regarding phasic pupil diameter changes, we are not confident that our results have provided ample evidence that phasic pupil diameter is sufficiently robust or reliable to guide automated competency assessments. When examining zoom-out behavior we did find evidence of a transient pupil dilatory effect immediately preceding a shift to low-magnification, presumably reflecting a shift from exploitation to exploration. However, this effect was not associated with the magnitude of the zoom event, the pathologists’ difficulty ratings of the image, the experience level of the pathologist, or whether they reached an accurate or inaccurate diagnostic decision. In other words, while phasic pupil diameter may be helpful for understanding the perceptual and cognitive processes engaged during the medical image interpretation, we have yet to discover ways that it will be helpful for guiding competency assessment during medical training. Continuing research will assess whether phasic pupil responses to very brief glances at biopsy images might reveal more interesting relationships with diagnostic accuracy.

## Conclusion

To our knowledge, we provide the first evidence that zoom behavior during a visual search task may partially reflect shifts of control states, perhaps indicating some aspects of adaptive LC-NE function. Naturalistic zooming behavior among pathologists interpreting biopsy images is exceedingly dynamic and complex, and we acknowledge that it is unclear whether discrete zoom-in or zoom-out events consistently reflect changes in control states. While we believe it is intuitive to think that a shift from low- to high-magnification zoom reflects a change of control states (exploration to exploitation), there are likely other reasons that pathologists zoom in. For example, some pathologists might zoom in to explore at a higher magnification (e.g., 5–10×) and employ a lawnmower-style search strategy [29] by panning in successive up-down or left-right paths across the image. In this case, a zoom-in event may reflect the adoption of a more fine-grained exploratory strategy, rather than a shift to exploitation. It is unlikely that there is a single threshold where participants discretely shift from exploration to exploitation; rather, it may exist on a continuum where, at times, both processes are engaged simultaneously (i.e., in parallel) or in rapid succession. Similarly, if pathologists do not see any compelling architectural features at low magnification to attract more focused attention, they might zoom in to continue their exploration. One of the challenges with collecting data during a naturalistic visual search task is the lack of control over such factors, motivating future work.

According to the AGT, shifting between exploration and exploitation states is associated with the constantly changing utility of a task. When a task is considered less rewarding (or rewards have been exhausted), we disengage from exploitation and return to exploring stimuli for higher rewards, but when new stimuli are encountered that may be more rewarding, we shift from exploration to exploitation. Given tight associations between LC-NE function and pupil diameter, pupillometry can prove valuable in examining shifts between control states. The present data provide mixed support for the application of AGT to continuous zoom behavior during a relatively naturalistic visual search task. While tonic pupil indeed dilated in association with increased interpretive difficulty of images, it also appeared to dilate with higher zoom levels, when pathologists are more likely in an exploitation rather than exploration state. Furthermore, when examining phasic pupil responses, we found that zoom-in events, which we proposed were indicative of shifts from exploration to exploitation, were associated with large phasic pupil constrictions. This was the opposite pattern than hypothesized based on AGT, and we present possible rationale for why this might be the case. Finally, when examining phasic pupil responses to zoom-out events, we found evidence of brief phasic pupil dilatory responses immediately preceding the zoom-out event, suggesting evidence of an adaptive exploitation-to-exploration shift of control states.
